# Supplementary material for: Content-rich biological network constructed by mining PubMed abstracts
Source: BMC Bioinformatics. 2004 Oct 8;5:147. doi: 10.1186/1471-2105-5-147 (PMC528731; doi:10.1186/1471-2105-5-147)
Supplement: Additional File 5 — The original Chilibot query results of the term "long-term potentiation (LTP)" and 22 other terms, limiting the latest references analyzed to the years 1990, 1995, 2000, and 2004. [file 1471-2105-5-147-S5.bz2 › chilibotAdditionalFile5/ltp2000/html/TRKA_NMDA.html]

 


 **TRKA** and **NMDA** 
  
Found 7 abstracts in PubMed,  **7 abstracts were retrieved and analyzed**.  


---

 Search Google  |
 PDF files only 
|  EDU domain only 

---

- J Biol Chem, 1998   **Activity dependent release of brain derived neurotrophic factor underlies the neuroprotective effect of N methyl D aspartate.** .
  The molecular mechanism s of N methyl D aspartate **NMDA** neuroprotective properties were investigated in primary cultures of cerebellar granule cell neurons.
  Granule cells express the neurotrophin receptor TrkB but not **TrkA** or TrkC.
  In these cells, the TrkB ligand brain derived neurotrophic factor BDNF prevents glutamate toxicity.
  Therefore, we have tested the hypothesis that **NMDA** activates synthesis and release of BDNF, which may prevent glutamate toxicity by an autocrine loop.
  Exposure of granule cells for 2 and 5 min to a subtoxic concentration of **NMDA** 100 microM evoked an accumulation of BDNF in the medium without concomitant changes in the intracellular levels of BDNF protein or mRNA.
  The increase in BDNF in the medium is followed by enhanced TrkB tyrosine phosphorylation, suggesting that **NMDA** increases the release of BDNF and therefore the activity of TrkB receptors.
  To examine whether BDNF and TrkB signaling play a role in the **NMDA** mediated neuroprotective properties, neurons were exposed to soluble trkB receptor IgG fusion protein, which is known to inhibit the activity of extracellular BDNF, and to K252a, a tyrosine kinase inhibitor.
  Both compounds blocked the **NMDA** mediated TrkB tyrosine phosphorylation and subsequently its neuroprotective properties.
  We suggest that **NMDA** activates the TrkB receptor via a BDNF autocrine loop, resulting in neuronal survival.

  - Proc Natl Acad Sci U S A, 1998   **Nerve growth factor rapidly suppresses basal, **NMDA** evoked, and AMPA evoked nitric oxide synthase activity in rat hippocampus in vivo.** .
    In adult forebrain, nerve growth factor NGF influences neuronal maintenance and axon sprouting and is neuroprotective in several injury models through mechanisms that are incompletely understood.
    Most NGF signaling is thought to occur after internalization and retrograde transport of **trkA** receptor and be mediated through the nucleus.
    However, NGF expression in hippocampus is rapidly and sensitively regulated by synaptic activity, suggesting that NGF exerts local effects more dynamically than possible through signaling requiring retrograde transport to distant afferent neurons.
    Interactions have been reported between NGF and nitric oxide NO .
    Because NO affects both neural plasticity and degeneration, and trk receptors can mediate signaling within minutes, we hypothesized that NGF might rapidly modulate NO production.
    Using in vivo microdialysis we measured conversion of L 14C arginine to L 14C citrulline as an accurate reflection of NO synthase NOS activity in adult rat hippocampus.
    NGF significantly reduced NOS activity to 61% of basal levels within 20 min of onset of delivery and maintained NOS activity at less than 50% of baseline throughout 3 hr of delivery.
    This effect did not occur with control protein cytochrome c and was not mediated by an effect of NGF on glutamate levels.
    In addition, simultaneous delivery of NGF prevented significant increases in NOS activity triggered by the glutamate receptor agonists N methyl D aspartate **NMDA** and alpha amino 3 hydroxy 5 methyl 4 isoxazolepropionic acid AMPA .
    Rapid suppression by NGF of basal and glutamate stimulated NOS activity may regulate neuromodulatory functions of NO or protect neurons from NO toxicity and suggests a novel mechanism for rapidly mediating functions of NGF and other neurotrophins.

    - Neuroreport, 1998   **BDNF gene transcripts in mesencephalic neurons and its differential regulation by **NMDA**.** .
      Brain derived neurotrophic factor BDNF enhances survival and protects dopaminergic neurons from neurotoxicity.
      We have examined in primary cultures of fetal mesencephalic neurons the expression of BDNF transcripts and its regulation by glutamate receptor agonists.
      RT PCR experiments showed in these cultures the expression of mRNA encoding for neurotrophin receptors **TrkA**, TrkB and TrkC as well as a differential expression of alternative BDNF gene transcripts derived from exons 1a, 1b, 1c and 1d.
      In adult rat mesencephalon we only detected BDNF mRNAs originated from exons 1a and 1c.
      Quantitative RT PCR analysis of mesencephalic neurons revealed that **NMDA**, but not kainate treatment, significantly increased BDNF mRNAs derived from exons 1b and 1d.
      Our data indicate the existence in mesencephalic neurons of different and alternative promoters within the BDNF gene that may be differentially regulated by glutamate receptor stimulation.

      - J Neurosci Res, 1997   **Cell death of adult pyramidal CA1 neurons after intraventricular injection of a novel peptide derived from **trkA**.** .
        Members of the nerve growth factor NGF family of neurotrophins bind to the second leucine rich motif LRM2 within the extracellular domains of their respective receptors **trkA**, trkB, trkC .
        Small LRM2 peptides have been recently demonstrated to selectively bind the neurotrophins revealing similar complex binding characteristics as full length receptors.
        We extend our recent findings, showing that the peptides A and C do not block nerve fiber outgrowth through high affinity trk receptors in a ganglia bioassay.
        Since the highest concentration of neurotrophins NGF, brain derived neurotrophic factor BDNF , neurotrophin 3 NT 3 is found in the hippocampus, the peptides were injected into the 3rd ventricle of anesthetized adult rats.
        The NGF binding LRM2 A peptide, but not the BDNF binding LRM2 B or the NT 3 binding LRM2 C peptides, caused severe apoptotic neurodegeneration of hippocampal pyramidal CA1 neurons as revealed by cresyl violet staining and the TUNEL reaction.
        The degeneration was protected by intrahippocampal injection of NGF beta and by the non N methyl D aspartate **NMDA** antagonist CNQX 6 cyano 7 nitroquinoxaline dione , indicating a glutamatergic mechanism.
        In situ hybridization revealed that pyramidal CA1 neurons did not express **trkA** and p75 receptor mRNA in sham and LRM2 A lesioned animals.
        It is concluded that the LRM2 A peptide represents a novel peptide with properties to induce apoptotic cell death of pyramidal CA1 neurons and may be useful as an experimental agent.

        - J Neurophysiol, 1997   Neurotrophin modulation of **NMDA** receptors in cultured murine and isolated rat neurons.
          J.
          Neurophysiol.
          78 2363 237 997.
          Patch clamp and calcium imaging techniques were used to assess the acute effects of the neurotrophins, brain derived neurotrophic factor , neurotrophin 3 , and nerve growth factor , on the responses of cultured and acutely isolated hippocampal and cultured striatal neurons to the glutamate receptor agonist N methyl aspartic acid .
          The effects of BDNF on **NMDA** activated currents were examined in greater detail.
          Currents evoked by **NMDA**, and the accompanying changes in intracellular calcium, were enhanced by low concentrations of the neurotrophins .
          The potentiation by the neurotrophins was rapid in onset and offset .
          The neurotrophins also reduced desensitization of these currents in most cells.
          The enhancement of **NMDA** activated currents by BDNF was observed using both perforated and whole cell patch recording techniques and could be demonstrated in outside out patches.
          Furthermore, its effects were not attenuated by pretreatment with the protein kinase inhibitors genistein or 1 2 methylpiperazine .
          Therefore, the actions of BDNF do not appear to be mediated by phosphorylation.
          Similar enhancements were observed with NT 3 and NT 4 and with NGF despite the fact that hippocampal neurons lack **TrkA** receptors.
          All together this evidence suggests that the enhancement of **NMDA** evoked currents is unlikely to be mediated through the activation of growth factor receptors.
          Modulation of **NMDA** responses by BDNF was dependent on the concentration of extracellular glycine.
          The most pronounced potentiation by BDNF was observed at low concentrations.
          . . . no potentiation was observed in saturating concentrations of glycine, suggesting that BDNF may have increased the affinity of the **NMDA** receptor for glycine.
          However, the competitive glycine site antagonist 7 chloro kynurenic acid blocked the enhancement by BDNF without shifting the dose inhibition relationship for this antagonist, and Mg2ion consistently depressed the potentiation of **NMDA** evoked currents by BDNF, indicating that BDNF does not alter glycine affinity.
          BDNF also reversibly increased the probability of opening of **NMDA** channels recorded from outside out patches taken from cultured hippocampal neurons.
          Other unrelated peptides including dynorphin and somatostatin also caused a glycine dependent enhancement of **NMDA** currents and depressed the currents in saturating concentrations of glycine.
          In contrast, a shortened analogue dynorphin , which lacks N terminus glycine residues, and another peptide met enkephalin were without effects on **NMDA** currents recorded in low concentrations of glycine.
          Our results suggest that neurotrophins and other peptides can serve as glycine like ligands for the **NMDA** receptor.

          - J Biol Chem, 1997   **Nerve growth factor up regulates the N methyl D aspartate receptor subunit 1 promoter in PC12 cells.** .
            The N methyl D aspartate **NMDA** subtype of glutamate receptor plays important roles in synaptic plasticity, the induction of long term potentiation, and excitotoxicity.
            Mechanisms governing the regulation of expression of its subunit genes remain largely unknown.
            The promoter of the essential subunit of the **NMDA** receptor heteromer, NMDAR1, contains DNA binding elements recognized by the nerve growth factor inducible early growth reaction factor NGFI Egr family of transcription factors that are rapidly induced by neurotrophins, such as nerve growth factor NGF .
            This study examined the effect of NGF on the activity of the N methyl D aspartate receptor subunit 1 NMDAR1 promoter luciferase reporter constructs in PC12 cells, which contain the high affinity **TrkA** receptor for NGF and the low affinity p75 NTR receptor for neurotrophins.
            NGF up regulated the activity of the NMDAR1 promoter by 3 4 fold in a time and dose dependent manner.
            5 deletional analysis of the promoter indicated that the responsive element s resides in the proximal region containing GSG and Sp1 sites.
            Mutational analysis of these sites revealed that both were important for NGF regulation.
            Transient expression of Egr 1 increased activity of the wild type promoter but failed to increase activity of a GSG mutant promoter.
            Other neurotrophins did not activate the promoter, while K 252a inhibited the action of NGF.
            These results suggest that the NGF effect is mediated by the high affinity NGF receptor, Trk A **[TRKA]**.
            neurotrophin binding to the low affinity neurotrophin receptor, p75 NTR , alone does not affect the promoter activity.
            Our results suggest that NGF is able to up regulate the activity of the NMDAR1 promoter and may play a role in controlling the expression levels of **NMDA** receptors.

            - Epilepsia, 1996   **Change in neurotrophins and their receptor mRNAs in the rat forebrain after status epilepticus induced by pilocarpine.** .
              We studied the effects of status epilepticus SE induced by lithium chloride pilocarpine treatment on gene expression of neurotrophins of the nerve growth factor NGF family and of their high affinity receptors of the tyrosine protein kinase trk family in the forebrain.
              Using in situ hybridization ISH , we demonstrated an early 3 h after treatment increase in brain derived neurotrophic factor BDNF and trkB mRNA expression in the dentate gyrus, amygdala, and piriform cortex, as well as widespread increases in the cerebral cortex.
              NGF mRNA, but not the mRNA of its receptor **trkA**, was increased in the dentate gyrus.
              In contrast, 12 h after treatment, neurotrophin 3 NT 3 decreased, and its receptor trkC mRNA increased.
              There was no change in NT 4 mRNA levels.
              All changes were blocked by pretreatment with scopolamine, a muscarinic antagonist.
              The noncompetitive N methyl D aspartate **NMDA** antagonist ketamine blocked NGF, BDNF, and trkB mRNA increases in the hippocampus and cerebral cortex, but not in the amygdala and piriform cortex.
              In contrast, ketamine did not affect NT 3 and trkC changes.
              These results provide a complete description of changes in mRNA levels of neurotrophins and their receptors in the forebrain after SE and supply additional data supporting the view that neurotrophin gene expression is related to abnormal neuronal activity.
